# Self-Perceived Quality of Life (WHOQOL-Bref), and Self-Reported Health, Social and Environmental Factors Related to Its Improvement among Residents of Anil, Rio de Janeiro—Cross-Sectional Study

**DOI:** 10.3390/healthcare12151520

**Published:** 2024-07-31

**Authors:** Rosemerie Barros, Alfredo Akira Ohnuma, Maria Conceição Manso

**Affiliations:** 1 Science and Technology Faculty, Fernando Pessoa University, 4249-004 Porto, Portugal; 34823@ufp.edu.pt; 2 Fisiocenter Recreio ltda, Rio de Janeiro 22790-381, Brazil; 3 Sanitary and Environmental Engineering Department, Faculty of Engineering, State University of Rio de Janeiro, Rio de Janeiro 20550-013, Brazil; akira@eng.uerj.br; 4 RISE-Health, Universidade Fernando Pessoa, Praça de 9 de Abril 349, 4249-004 Porto, Portugal; 5 Faculty of Health Sciences, Universidade Fernando Pessoa, Rua Carlos da Maia, 296, 4200-150 Porto, Portugal; 6 FP-I3ID (Instituto de Investigação, Inovação e Desenvolvimento), FP-BHS (Biomedical and Health Sciences), Universidade Fernando Pessoa, Praça de 9 de Abril 349, 4249-004 Porto, Portugal

**Keywords:** WHOQOL, WHOQOL-bref, QoL, health promotion, socio-environment, sustainability

## Abstract

This study aims to assess the self-perception of the QoL (WHOQOL-bref) in the Canal of Anil zone and its neighbor zone of the center of the District of Anil in Rio de Janeiro and to identify which factors are associated with the population self-perception of the need to “improve” their quality of life (QoL). A cross-sectional observational analytical study was carried out after approval by the competent ethics committee (CEP/CONEP) approval. A non-probabilistic sampling of residents of the Canal of Anil (n = 494) and the central district of Anil (n = 250) was used. A questionnaire was administered in person to collect data on self-reported sociodemographic characteristics, general health, sanitation, lifestyle in the residential area, and the WHOQOL-Bref. Although with a worse self-perceived water/sanitation participants in the Anil Canal community report fewer allergies, less medication, fewer skin diseases, less Zika virus, and less Chikungunya, among others. The self-perception of the need to improve the QoL in the Anil Canal community and the zone at the central District of Anil has proved to be influenced by several social and economic factors as well as residential practices and conditions. The multivariate analysis allowed us to identify both modifiable and non-modifiable risk factors for the need to improve physical QoL: taking medication, respiratory problems, skin disease diagnosed by a doctor, having a water tank at home or having filtered water at home, unpleasant odor of the water of the Anil Canal and the level of education, and age. Regarding the need to improve the environmental QoL, both areas are largely modifiable (e.g., having had ascariasis/roundworm; having a water tank in the house; not drinking bottled water; not having pavements in the street). Sociodemographic and environmental factors, in addition to health conditions, play a pivotal role in influencing individuals’ perceptions of the necessity for enhanced physical and environmental well-being.

## 1. Introduction

The concept of quality of life has been defined by several specialists as the perception of individuals of their position in life in the context of the culture and value systems in which they live and their aims, expectations, standards, and concerns [1]. It is a wide-ranging concept that encompasses the complexity of the construct and is linked with the environment through physical and psychological factors, as well as a level of independence, social relationships, and personal beliefs [2]. There are several studies that assess the quality of life of people in a particular age group [3], usually just the elderly [4,5], but those that cover most of the (adult and elderly) community are rarely found or non-existent. The study for women from a quilombola community in northeastern Brazil [6] or the quality of life and urban environment in São Paulo city [7] does not include a survey or assess the adult and elderly community within a major city. 

Assessing the quality of life of a community can be carried out through instruments such as the WHOQOL (World Health Organization Quality of Life) [8] or in its shorter version the WHOQOL-Bref [9]. The WHOQOL-Bref comprises four dimensions: physical dimension, psychological dimension, social relations, and environmental dimension [9]. The WHOQOL-Bref instrument is favored for its comprehensive, efficient, and culturally adaptable approach to measuring quality of life. Its design ensures it captures a wide range of life domains, making it a valuable tool for both research and practical applications in diverse settings [9]. Its advantages over similar instruments include its brevity, holistic coverage, and global applicability. Moreover, the WHOQOL-Bref has already been translated, adapted, and validated for the Brazilian population [10].

The epidemiological investigation of a health issue allows for the implementation of preventative measures regarding the health-disease process and the geographic and environmental features of a particular place. Human health is influenced by climate conditions [11]. It is of the utmost importance to implement environmental sanitation practices in order to foster the quality of life. These practices serve to control the physical surroundings with the aim of preventing the occurrence of diseases and ensuring greater social hygiene. Services for the supply of drinking water to the public, the collection system, transport and final waste disposal, sewage collection and treatment systems, and urban rainwater drainage are essential for the required conditions of the health and wellbeing of the public [12,13]. Social factors such as income and employment, education, cultural factors, and crime and safety have an impact, as well as environmental factors, e.g., the housing quality, the access to healthcare facilities, availability of parks and recreational areas, air and water quality, and the integration social–environmental, as the urban planning and social equity, environmental education, and effective local governance that integrates social policies with environmental management can improve the overall quality of life. Poor health conditions in the environment or surrounding atmosphere can make people feel worried and anxious, and this can impair their perception of the quality of life. The poor quality of the air or water, for example, can cause health problems such as respiratory diseases and skin infections. In addition, poverty and inequality can aggravate health and environmental problems further and lead to an even lower quality of life. Inadequate access to basic services such as drinking water and sanitation can also impair the perception of the quality of life since these are essential requirements to maintain good health. 

This study seeks to assess the self-perception of the QoL (WHOQOL-bref) in the Canal of Anil zone and its neighbor zone of the center of the District of Anil in Rio de Janeiro, as well as to identify which factors are associated with the population self-perception of the need to “improve” the quality of life (QoL).

## 2. Materials and Methods

### 2.1. Study Type and Ethics’ Concerns

An analytical, observational, and cross-sectional study design was conducted. This study was approved by the CEP/CONEP [Committee of Ethics/National Council of Ethics in Research] system through the Brazil Platform (Approval Number: 2.620.525, CAAE [Certifying Statement by the Critical Ethical Review] 74415017.2.0000.5282 approved on 25 April 2018).

### 2.2. Sample and Sampling Size

**Figure 1:**
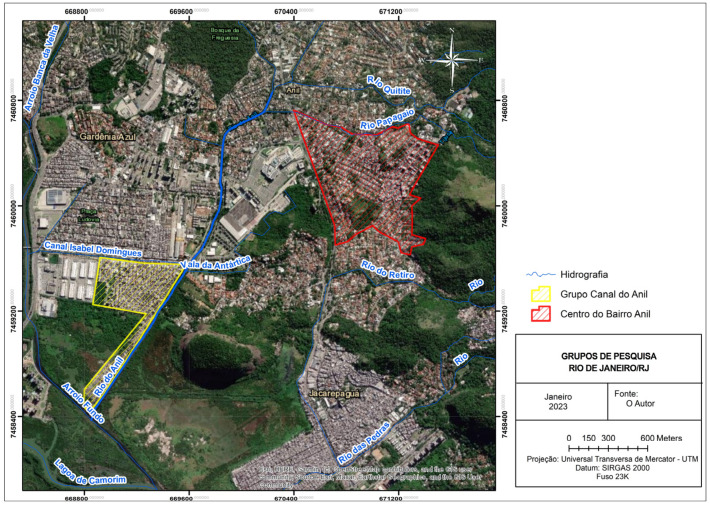
Image of the two units of analysis that were of interest to the Anil community. Yellow area (left side) represents the Anil canal area, red area (to the right side) represents the center of the District of Anil, and the blue lines represent both the Canal do Anil microbasin and the area’s hydrography in the region close to the Canal of Anil, Rio de Janeiro. Source: GOOGLE EARTH 2023 (Image generated in ArcGIS 10.3).

The sampling plan comprised two units of analysis that were of interest to the Anil community (in the lower region of Jacarepaguá), RJ, Brazil from June 2018 to June 2019 by applying a questionnaire in a single period (Figure 1). 

The demographic areas described in the two study zones are in almost contiguous areas of the same neighborhood (Anil), located in the city of Rio de Janeiro. The Anil canal area was chosen because it qualifies as an area described in the municipality of Rio de Janeiro as a “comunidade”, namely an area where most residents have a family income below or equal to the minimum wage. This community (“comunidade”) is characterized by substandard housing (a mix of “favela” (informal settlements that are often characterized by overcrowding, lack of basic infrastructure, and inadequate access to services) with irregular settlement), bad sanitation conditions, and is exposed to environmental determinants. The other study area is represented by the population in the center of the District of Anil who has a higher family income and has their own housing and sanitation conditions. This represents a kind of a control group, as it is not exposed to the same social and environmental factors. Consequently, the cross-sectional study is situated in the same neighborhood and province, with two zones that are geographically proximate but exhibit considerable divergence.

The sample size was determined based on the size of the population of the District of Anil, which after consulting the Census of 2010, was approximately 24,152 people [14]. For the calculation, it was assumed that 50% of the sample had at least a “good” QoL (or 50% saying the opposite), a level of significance (α) of 1%, and a confidence interval width of ±5%, which resulted in the sample size of the group in the Canal of Anil, including at least 462 adults. In the case of the other area (the center of the District of Anil), given a 95% confidence interval (α = 5%) and the same specific estimates and width referred to earlier, the sample should attain at least 244 adults. The inclusion criteria in the sample were as follows: being a resident in the area under study and being 18 years old or older. Residents under 18 years old were not included as well as non-residents. 

The sample (non-probabilistic sampling method) of this epidemiological study consisted of residents of both sexes who were at least 18 years old in the Anil canal area (n = 494) and in the center of the District of Anil (n = 250).

### 2.3. Data Collection

The questionnaire was given face-to face by the first author. It includes close-ended questions that cover self-reported sociodemographic data (in which marital status was classified as: single/married/divorced/widowed; schooling as: illiterate/basic/secondary/higher–both variables were considered potentially confounding factors of the results), self-reported general health information, self-reported concerns about water and sanitation in the residential areas (mostly assessed for the “last year period”), the lifestyle, and the assessment of the self-perception of the QoL by means of the WHOQOL-bref [10]. 

Income was categorized as one, two to three, and at least four minimum wages. Brazilian law provides for a minimum wage for workers with a formal contract so most Brazilian states follow the value set by the federal government, with the minimum wage in 2018 being set at BRL 1136.53 (approx. 238 US dollars).

The WHOQOL-bref instrument comprises 26 questions divided into four domains: physical, psychological, social relationships, and the environment—in this study, the emphasis was on the results emerging from “QoL physical factors” (Domain 1) and “QoL environment” (Domain 4). The results of the self-perception of the QoL (%)—the higher the percentage (the closer to 100%), the better the quality of life—were subsequently divided into the following categories: (i) “need to improve” (the average result of the domain from 1 to 2.9), (ii) “regular” (3 to 3.9), (iii) “good” (4 to 4.9), and “very good” (5) [10]. For the purposes of analysis regarding the QoL results, because it was observed that not all the QoL domains showed differences between the two study zones (depicted in the paper results), it was decided that the domain where the most pronounced divergence was observed, the environmental domain would be the focus of the analysis. However, it was also considered beneficial to select one additional domain (of the remaining three) for analysis to obtain a more concise result section. The remaining domain with the greatest disparity (although without significant differences between the two zones) was the physical domain. The potential for relationships between the physical domain and health or household issues was another factor that influenced our decision. The “QoL physical domain” and “QoL environment domain” were subsequently dichotomized as “need to improve” and “no need to improve (regular, good, very good)” to identify risk/protection factors for the “need to improve” QoL of these two domains in both study areas.

### 2.4. Data Analysis

The statistical analysis procedures were carried out with the aid of IBM^®^ SPSS^®^ Statistics vs.25.0 software (IBM Corp. Released 2017, Armonk, NY, USA: IBM Corp.). The fixed level of significance was 0.05. 

Nominal and ordinal qualitative data were described for counts and respective percentages (n, %). Median statistics were used and the respective interquartile range (showing the 1st and 3rd quartile (Q1; Q3)), and the range (minimum and maximum) was observed for the quantitative variables because data normality had not obtained (that is, assessed by the Kolmogorov–Smirnov (n ≥ 30) or Shapiro–Wilk tests (n < 30). The average and standard deviation (SD) are shown to allow a comparison to be made with the information in the scientific literature.

The comparison between the nominal and ordinal qualitative variables in the two groups (the Anil canal area and the neighboring area of the center of the district of Anil) was carried out with Chi-Square tests. Since normality in the distribution of the quantitative variables was not obtained, the comparison between the two independent groups was carried out by the Mann–Whitney U tests, and in the case of more than two independent groups, by Kruskal–Wallis tests. The self-perception of QoL was analyzed in accordance with the recommendations of Fleck et al. [10] and in http://www.cefid.udesc.br/arquivos/id_submenu/1173/whoqol_bref.pdf (accessed on 19 September 2019).

Alpha of Cronbach coefficient was calculated for the two zones being studied, which are the WHOQOL-bref instrument and the four dimensions in order to determine the internal consistency of these sets of survey items. 

The association between the ‘need to improve the physical domain of QoL’ and ‘need to improve the environmental domain of QoL’ and the relevant covariates (bivariate analysis) was assessed using the unadjusted odds ratio (OR). Afterward, there was an assessment of the independent effect of the variables or significant co-variables (*p* < 0.05) on the result of the physical domain and, separately, in the environmental domain of the QoL questionnaire (in both cases for the “need to improve” category). This was made through stepwise multivariate analysis of a binary logistic regression (0.05 for the inclusion of co-variables and 0.2 for their exclusion) and then adjusted. The multivariate models of the result for “the need to improve the physical domain of QoL”, and the “the need to improve the environmental domain of QoL” include socio-demographic variables and those concerned with health and information about water and sanitation in the area. The assessment of the multivariable models relied on the following test criteria: (i) 2loglikelihood, (ii) Cox and Snell coefficient correlations, (iii) the Nagelkerke test, and (iv) the Area Under the Curve (AUC) derived from the model after conducting a ROC analysis.

## 3. Results

In this study, 494 people were initially questioned in the Anil Canal area and 250 in the center of the District of Anil. As not all the questions were answered for the WHOQOL-bref questionnaire, the results of some of the participants had to be excluded because the QoL questionnaire result is only valid if at least 80% of the 26 questions were replied. Thus, results are shown for the participants where it was possible to analyze the questionnaire on the QoL or in other words, 485 in the Anil Canal area community (n_excluded_ = 9) and 249 in the center of the District of Anil (n_excluded_ = 1). For these 98.2% and 99.6% of the respondents, there were further questions that they decided not to answer but no more participants were excluded for that reason, as it can be found throughout the presentation of the results.

The alpha of the Cronbach coefficient was 0.865 and 0.871 for the WHOQOL-bref instrument in the Anil Canal area and in the center of the District of Anil, respectively. For both zones and the four domains, we obtained, respectively, 0.435 and 0.485 for the physical domain, 0.573 and 0.606 for the psychological dimension, 0.603 and 0.608 for the social relations domain, and 0.809 and 0.821 for the environmental domain. 

Regarding the socio-demographic features of the residents in the two zones (Table 1), it was possible to determine that there was no significant difference in the distribution by sex (Chi^2^-T, *p* = 0.890) and in the median residence time in the local area (20 years; Mann–Whitney U Test, *p* = 0.727) in both groups. The participants from the Anil Canal area were significantly younger than in the center of the District of Anil (median age 43 vs. 49 years; Mann–Whitney U Test, *p* < 0.001), include a significantly smaller number of ethnically white people (25.5% vs. 44.0%), and include more who were ethnically brown (61.7% vs. 43.0%) than those questioned in the center of the District of Anil (Chi^2^-T, *p* < 0.001). Moreover, there were significantly more single people (46% vs. 36.9%), fewer divorced (29% vs. 8.2%), and fewer widowed (4.5% vs. 9%) than among those questioned in the center of the District of Anil (Chi^2^-T, *p* < 0.001). Within the Anil Canal area, significantly less people were born in Rio de Janeiro when compared to the center of the District of Anil (49.3% vs. 70.3%; Chi^2^-T, *p* < 0.001).

Regarding the level of educational attainment (Table 1), significant differences were detected in the two groups—basic education was significantly higher in the Anil Canal area (37% vs. 27.5%) and higher education was significantly higher in the center of the District of Anil (11.7% vs. 24.8%). This fact might be attributed to the difference in income that was found in the two groups, where only one minimum salary was more prevalent in the Canal of Anil Community (67.5% vs. 46.6%) and 2–3 minimum salaries or at least 4 minimum salaries were more common in the center of the District of Anil (Chi^2^-T, *p* < 0.001).

With regard to information about health (Table 2), the differences noted in the two groups (the Anil Community when compared with the central District of Anil) are due to the rates being significantly lower in the following: the cases of allergies (35.7% vs. 47.8%; *p* = 0.001), use of medicines (29.2% vs. 42.9%; *p* < 0.001), having an annual check-up (40.8% vs. 56.3%; *p* < 0.001), a skin disease diagnosis (8.5% vs. 13.8%; *p* < 0.001), less Zika (1.6% vs. 5.2%), and less Chikungunya (2.1% vs. 6.4%; *p* = 0.002). In addition, the self-perceived psychological health is worse (*p* = 0.013) because there are significantly fewer cases that can be categorized as excellent (44.5% vs. 57.4%) and more that are categorized as normal (49.2% vs. 38.4%).

Nearly universal access to public pipeline water in both areas (98.8% in the Anil Canal area and 99.2% in the Central District). The results showed more households in the Central District have water filters (93.5%) compared to the Anil Canal area (83.6%) (*p* < 0.001) and fewer reports of smelly drinking water in the Central District (5.2%) than in the Anil Canal area (12.2%) (*p* = 0.003). None of the Central District residents use Anil Canal water for any purposes, compared to 9.9% in the Anil Canal area (*p* < 0.001). Children playing in Anil Canal is less common for residents in the Central District (48%) compared to the Anil Canal area (75.2%) (*p* < 0.001). The unpleasant smell from Anil Canal was more frequently reported by residents in the Anil Canal area (89.9%) than in the Central District (76.8%) (*p* < 0.001), as well as the regularity of this smell as a daily problem, 68.2% vs. 53% (*p* < 0.001). The flooding near the residence was more frequent in the Anil Canal area (88.6%) than in the Central District (58.6%) (*p* < 0.001), and the same pattern was observed for garbage accumulation (63.5% vs. 35.9%; *p* < 0.001) but no significant difference was found about the local garbage collection (*p* = 0.180).

In generic terms, the QoL and satisfaction with one´s health (Table 2), do not differ significantly between the Anil Community and the Central District of Anil (*p* = 0.391 and 0.235, respectively). Moreover, no significant difference was discovered when there was an assessment of (i) the self-perception of the QoL regarding physical questions (Domain 1—Physical; *p* = 0.956), (ii) psychological matters (Domain 2—Psychological; *p* = 0.647), and iii) social relations (Domain 3—Social Relations; *p* = 0.972). The difference detected is only significant in regard to the environmental domain (*p* < 0.001) as it is significantly worse in the Anil Canal Community with a need for improvement of 71.5% in comparison with 43% in the center of the District of Anil. This is the only one of the 4 domains where nobody achieved a higher level of satisfaction (i.e., ‘very good’) in their self-perception. 

These results are also supported by further evidence when they were compared through the other system of measurement that the questionnaire allows us to use–the percentage scale–both presented in Table 2. The QoL associated with the environment is self-perceived in a worse way in the Anil Community than in the Central District of Anil (40.6% vs. 50.0%; Mann–Whitney U Test, *p* < 0001). The other 3 domains for the QoL do not show significant differences in the two zones assessed (*p* > 0.05). 

There is a significant positive relation between QoL (i.e., each of its four domains) and self-perceived physical health, showing that the worse physical health is self-perceived, the worse the QoL is also self-perceived. As physical health shifts from excellent to normal or from poor to terrible, the percentage corresponding to the self-perception of the QoL also becomes lower and this happens in the same way for both the Anil Canal Community and the Central District of Anil zone (*p* < 0.001). In Domain 4 (Environment), the percentage of self-perception for the QoL is significantly lower for excellent and normal physical health (*p* < 0.001) in the Anil Canal community than in the Central District of Anil zone but there is no significant difference in the two areas if the physical health is self-perceived as impaired (poor or terrible; *p* = 0.274).

Table 3 depicts the univariate and logistic regression multivariate analysis of risk/protective factors linked to the resulting “need to improve” the physical domain of the QoL both at the Anil Canal community and at the Central District of Anil. 

The multivariate analysis shows that in the Anil Canal community the factors that are closely linked to the “need to improve” the QoL in the physical domain (Table 3) are as follows: (a) the marital status, being widowed is a risk factor (OR ≈ 11.414 (95%CI: 3.770–34.554)) while being married is a protective factor (OR ≈ 0.526 (95%CI: 0.282–0.980)) compared to being single; b) currently taking any medicines (OR ≈ 3.2 (95%CI: 1.8–5.8)); (c) having respiratory problems (OR ≈ 3.6 (95%CI: 1.9–6.8)); (d) having filtered water in the house (OR ≈ 3.3 (95%CI: 1.7–6.4)); (e) the Anil Canal has an unpleasant smell (OR ≈ 4.9 (95%CI: 1.1–22.5)). Regarding the zone in the center of the Anil District, there is an equal number of factors that are significantly associated with the “need to improve” the QoL in the physical domain (Table 3) but they are partially different. Thus, the determined risk factors include age (for each year getting older the odds of a “need to improve” for the physical QoL increase by 1.034 (OR ≈ 1.034 (95%CI: 1.005–1.064)). Level of education has a higher level at 5.3 higher odds for that outcome (OR ≈ 5.3 (95%CI: 1.5–19.5)) and the lower levels of education without significant odds and effect. Other risk factors are currently taking medicines (OR ≈ 3.4 (95%CI: 1.3–9.0)), having been diagnosed with any skin diseases (OR ≈ 6.5), and not having a water container/tank in the house (OR = 1/0.192 ≈ 5.2 (95%CI: 1.1–25.6)). 

It could be determined from the multivariate analysis the risk factors that are closely linked to the “need to improve” the QoL in the Environment domain (Table 4) in the Anil Canal community. They are as follows: (a) low income [in the case of people who earn a minimum salary there is a ten times greater risk of a need to improve the “environment” compared with those who earn 4 or more minimum salaries (OR ≈ 10.2 (95%CI: 2.2–46.5)) and in the case of those who earn 2–3 minimum salaries, there is about a 6.7 times greater risk of the need to improve the “environment”, compared with those who earn 4 or more minimum salaries (OR ≈ 16.7 (95%CI: 1.4–30.9)); (b) people who have never had ascariasis/roundworm (OR ≈ 2.5 (95%CI: 1.2–5.2)); (c) people who have a water container or tank in their house (OR ≈ 3.1 (95%CI: 1.2–8.1)); (d) the water that is drunk is not bottled (OR ≈ 2.1 (95%CI: 1.2–3.7)); (e) there are no pavements in the zone where the house is located (OR ≈ 2.1 (95%CI: 1.2–3.7)); (f) there is an accumulation of garbage (OR ≈ 2.6 (95%CI: 1.5–4.5)).

Regarding the central of the Anil District, there are fewer factors that are significantly associated with the “need to improve” the QoL in the Environment domain (Table 4) and although some are similar to the Anil community, they are in less number. Thus, the risk factors include: (a) low income [in the case of those who earn a minimum salary, there is around a 5.6 times greater risk of the need to improve the “environment” compared with those who earn 4 or more minimum salaries (OR ≈ 5.6 (95%CI: 1.9–16.5)) and feel this need. In the case of those who earn 2–3 minimum salaries, this entails an increased risk of about 3.3 times the need to improve the “environment” compared with those who earn 4 or more minimum salaries (OR ≈ 3.3 (95%CI: 1.1–9.8)) and feel this need for improvement]; (b) there are no gutters or sewer-grates for the drainage of rainwater in the zone where the house is located (OR ≈ 1.8), compared with people who have these facilities and do not feel this need; (c) there is an accumulation of garbage (OR ≈ 3.0 (95%CI: 1.7–5.3)), compared with those who do not feel this need for improvement.

## 4. Discussion

This study on QoL is not the only one made with a Rio de Janeiro sample as there are studies about caregivers of visually impaired children [15], QoL among people living with HIV/AIDS [16], QoL of primary care patients [17], people treated for tuberculosis and HIV [18], the relationship of alcohol consumption and mental disorders of patients in primary health care [19], people with schizophrenia [20], medical students [21], people with multiple sclerosis [22], adolescents with asthma [23], and QoL impacted by traditional Chinese medicine techniques [24], among others, but to our best knowledge this study is the only one using as a sample a community of people having no specific disease or condition. Moreover, this study carried out in the city of Rio de Janeiro (Brazil) quantified the QoL of two neighboring areas, one more deprived (Anil Canal community) and the other with better social conditions (central District of Anil).

Contrary to the environmental QoL domain, the physical, psychological, and social QoL domains showed no significant differences between study areas (Table 2), though the differences can still be relevant. The WHOQOL-bref tool might not detect subtle differences and many individuals spend substantial time in both areas due to work, family, or social reasons, leading to similar QoL outcomes. Factors like coping mechanisms, resilience, strong community ties, and support networks can maintain psychological well-being even in more polluted areas. Social connections and relationships might be equally strong, with cultural norms and shared events promoting social cohesion and enhancing social QoL equally in both areas.

The present study dealt with the identification of factors associated with changes in the QoL perceived by the population of the Anil district (two different areas), particularly for modifiable factors related to the physical aspect of quality of life and environmental structure. No study displays self-perception of the need to improve the QoL based on the four domains, including physical, psychological, social relationships, and the environment, in two different locations in the same district of the same city. Another novelty and unique feature of this study is that although with some limitations and although the overall perceived QoL was not different in both areas of the same district (Anil district), it was possible to understand that the QoL related to the environment domain was significantly different. Moreover, while with no significant differences in the physical domain of the QoL for both areas, some similar factors (currently taking any medicine and having filtered water in your house) but also different factors were associated with the need to improve it in both zones. Later, regarding the most different domain, the environment, it was also possible to attain that some differences were perceived, therefore, the variables associated with the need to improve the environment domain of QoL were not always the same for both areas (water container/tank in your house, not drinking bottled water, and no pavement in the street outside the house were factor affecting the Anil Canal area). When they were found to be similar, in some cases, the impact of the variables was almost double in the Anil Canal area (income, as an example) and others had almost the same impact in both areas (garbage accumulation around the house). 

It was determined that ´marital status´ was a factor involved in the self-perception of the need to improve the physical QoL in the Anil Canal community (since being widowed is a risk factor (OR = 11.4) in the wish to improve this aspect of the QoL and being married is a protective factor (OR = 0.526)) which suggests that marital status plays a role in the perception of the physical QoL. This is in line with what has been reported about the widowed state by Campolina and colleagues [24] who applied the SPF-12 test but contradicts the findings about the marriage/non-marital partnerships because in her study (which was conducted in São Paulo), being single was shown to have a higher QoL. Further risk factors that were found were the use of some medicines (OR ≈ 3.2) or respiratory problems (OR ≈ 3.6), which is also partially confirmed in the previously mentioned study [25] about recent health complaints or illnesses in the past. Risk factors were also detected that were related to infrastructural problems such as the question of having a water filter in the house (OR ≈ 3.3) which suggests that clean water can have a positive influence on the perception of the physical QoL. At the same time, the sense that the Anil Canal water has an unpleasant smell (OR ≈ 4.9) was linked to a greater probability of a desire to improve the physical QoL. Although these last two factors are not mentioned in other studies on the QoL in urban Brazil, both the necessity and appropriateness of using water of good quality in rural zones are both cited and validated by Neves-Silva and colleagues [26]. 

In the Central District of Anil, the factors linked to the self-perception of the need to improve the physical QoL were as follows: (i) aging (OR ≈ 1.03) through an increase of one year and (ii) having a higher level of education (OR ≈ 5.3). Both are potentially linked to data awareness through the experience and the access to other localities, as well as the use of some kind of medicine (OR ≈ 3.4), and the fact that a doctor has diagnosed a skin disease (OR ≈ 6.5). This suggests that people who take medicine or have skin problems may have underlying health conditions or symptoms that might affect their desire for improvement, and furthermore, are related to a perception of a loss of QoL. 

At the same time, having a water container or tank in the house operates as a factor that does not increase, e.g., that protects from the need to improve the quality of physical life (OR ≈ 0.19), since this result suggests that having access to stored water can lead to a more positive perception of the quality of physical life. 

The protection of the environment and the QoL are matters of extreme importance for the well-being of communities. Despite the seemingly worse self-perceived water/sanitation, according to Table 1, participants in the Anil Canal community report fewer allergies, less medication, fewer skin diseases, less Zika virus, and less Chikungunya, among others, which was unexpected. No statistical reason was highlighted from the results but one can think individuals who are more resilient or have stronger immune systems (as they are significantly younger) might be the ones who continue to live in environments with poor water and sanitation (no significant difference in living time in the two areas was observed). This could lead to a form of survival bias where those who are more susceptible to health issues have already moved away or have experienced adverse outcomes not captured in the study. One might hypothesize that this phenomenon is related to the lack of awareness of the disease or the reluctance to acknowledge it.

The self-perception of the need to improve the environmental QoL in the Anil Canal community and the zone in the central District of Anil has proved to be influenced by several social and economic factors, as well as residential practices and conditions. This is in accordance with or may be partially explained by the changes resulting from the disorderly occupation process since 1970 have led to countless transformations in the Anil Canal region, with a high rate of population growth in the area [27]. This urban sprawl has led to environmental degradation and consequently an increase in the fragility and risks of flooded areas that can cause health problems, basic sanitation, and poor quality of life [28,29].

Thus, in the Anil Canal community, it was found that the lower the income the greater this perception of the need to improve (OR ≈ 10.2 in the case of those who only earn 1 minimum salary and OR ≈ 6.6 in the case of those who earn 2–3 minimum salaries, compared with those who earn 4 or more). These results can be explained by the greater socio-economic vulnerability of people with a low income. They often face more precarious living conditions and suffer from a lack of access to basic services and inadequate facilities, which makes them more aware of the environmental problems that affect their lives [30,31]. In addition, if people have not had ascariasis or roundworm, this can be viewed as a risk factor (OR ≈ 2.5), as it suggests that the need to maintain personal health can influence the perception of environmental problems. Having a water container or tank in the house (OR ≈ 3.1) and not drinking bottled water (OR ≈ 2.1) suggest that having access to a reliable source of water can give rise to the perception that the environmental QoL is better and more under control and thus confirms that environmental awareness might be linked to a preference for sustainable sources of water. Not having a pavement outside the house (OR ≈ 2.1) and having accumulated rubbish in the surrounding area (OR ≈ 2.6) suggest that cleanliness in the urban infrastructure might play a key role in the way the local inhabitants perceive environmental problems. 

With regard to the central District of Anil, the factors linked to the self-perception of the need to improve the environmental QoL also included the income but it is important to note that although the confidence intervals of these categories in both areas overlap (revealing no significant differences in the impact), the estimate of the OR is about half the one of the Anil Canal area. The other similar factor is the accumulation of rubbish around the house (OR ≈ 3.0) with almost the same estimate of impact. These results underline the importance of addressing the problem of socio-economic inequality and improving the living conditions of the most vulnerable communities, as well as seeking to foster a heightened awareness of this and encourage greater involvement in the search for an improvement in the quality of their environmental life. Policy-making that takes account of these factors is essential to bring about positive changes and environmental sustainability in these areas. The involvement of the governments or local authorities in educational campaigns and their participation in decision-making about environmental issues is also essential for creating a society that is more conscious and responsible regarding the environment.

Among the risk factors for physical QoL, those associated with health are moderately modifiable. These include taking medication at the moment, having a respiratory problem, and having a skin disease diagnosed by a doctor. Also, modifiable/alterable are those associated with housing or local conditions. These include having a water tank at home or having filtered water at home. The unpleasant odor of the water in the Anil Canal can be altered over time, as can the level of education. In some cases, marital status may be modified, but age, which has been identified as a significant risk factor in the population of the center of Bairro do Anil, is a non-modifiable risk factor.

The risk factors for the need to improve the environmental QoL in the Canal do Anil community and in the central area of Bairro do Anil are largely modifiable (e.g., having had ascariasis/roundworm; having a water tank in the house; not drinking bottled water; not having pavements in the street). Policies aimed at improving health and housing conditions could potentially alter the prognosis. In contrast, changes in economic income are more challenging to implement in the short term, although they cannot be considered non-modifiable. 

Some risk/protective factors can be modified within the context of public health policies. However, for this to occur, governments or local authorities must implement public health policies that are oriented towards disease prevention and health promotion. These policies must also encompass infrastructure and urban planning, as well as health education, which should provide information on topics such as nutrition, physical exercise, disease prevention, and stress management, as well as access to health services. This will enable individuals to make healthier choices in their daily lives.

### Limitations to Generalization

It is important to note that this study, like all research, has limitations. A method of convenience sampling was employed and the residents who took part were largely people who spent more time in the two areas, while those who had spent most of their time away from the house had less chance of being involved. Although all data were obtained in a self-reported manner, and memory bias can occur, it should be made clear that it is extremely difficult to make direct contact with people living in deprived neighborhoods where the interviewer is an unknown person, and this fact can even entail risks for the interviewer. Nevertheless, as we found that the length of time spent living in the respective areas did not differ significantly, the previous fact may not have affected the results and thus was not used as a control variable for the effects of residence on the perception of the QoL.

It should be considered that the results are based on a cross-sectional study through which it was not possible to determine a cause-effect and temporal relationship between the independent variables and QoL, even though this was not the objective of this study.

Several other factors were not investigated, such as residential satisfaction, community feeling, and family relations in the neighborhood, which might have a mediating effect on the environmental QoL of the vicinity. 

Despite those limitations, valuable lessons can be learned from the results of this study since they supply a useful summary and key evidence of the links between the urban environment and the QoL in a Brazilian urban area.

## 5. Conclusions

The results of the study highlight the fact that there is a complex interaction of a wide range of factors that influence the self-perception of a need to improve the QoL of the residents of Anil (Canal of Anil and the center of the district) in Rio de Janeiro. Some of these factors are modifiable. They involved socio-economic and socio-environmental factors, namely those related to the following: marital status, level of education, income, waterborne diseases, environmental sanitation, public drinking water supply services, collection systems, transport, solid waste disposal, wastewater collection and treatment, and urban rainwater drainage systems, all of which are essential for the health and wellbeing of the public. 

Based on the findings obtained in this self-reported QoL assessment, it would be recommendable to pursue intervention-based research, aimed at improving specific modifiable factors such as environmental sanitation, public drinking water supply, and wastewater treatment and evaluate their effectiveness. Moreover, another way could be to perform comparative studies between Anil and other similar districts to identify best practices and effective strategies that could be replicated to enhance QoL in similar areas.

In essence, the implementation of alterations by political or municipal authorities can engender an enabling of physical and environmental factors conducive to the adoption of healthy behaviors and the modification of risk factors, thereby fostering an enhancement in the population’s QoL. 

## Figures and Tables

**Table 1:** Characteristics of the sample in the Anil Canal area (n = 485) and the central District of Anil (*n* = 249).

Variables/Factors		Anil Canal Area	Central District of Anil	*p*
**Socio-Demographic Information**				
Sex	F, n (%)	303 (62.6%)	159 (63.9%)	0.739
Age (years) (n_CA_ = 481/n_CD_ = 245)	Median (Q1; Q3)	43 ^b^ (34; 56)	49 ^a^ (36; 63)	**<0.001**
	Min-Max	18–100	18–87	
	average (SD)	44.2 (15.1)	49 (17.1)	
Ethnic origin * (n_CA_ = 345/n_CD_ = 200)	white, n (%)	88 ^b^ (25.5%)	88 ^a^ (44%)	<0.001 *
	black, n (%)	44 (12.8%)	24 (12%)	
	brown, n (%)	213 ^a^ (61.7%)	86 ^b^ (43%)	
	mixed Indian and European, n (%)	-	1 (0.5%)	
	mixed African and European, n (%)	-	1 (0.5%)	
Marital status (n_CA_ = 448/n_CD_ = 244)	single, n (%)	206 ^a^ (46%)	90 ^b^ (36.9%)	**<0.001**
	married, n (%)	209 (46.7%)	112 (45.9%)	
	divorced, n (%)	13 ^b^ (2.9%)	20 ^a^ (8.2%)	
	widowed, n (%)	20 ^b^ (4.5%)	22 ^a^ (9%)	
Residence time in the area (number of years) (n_CA_ = 441/n_CD_ = 245)	Median (Q1; Q3)	20 (10; 35)	20 (10.5; 31.5)	0.727
	Min-Max	0.3–75	0.2–60	
	Average (SD)	22.6 (15.0)	21.8 (13.3)	
Place of birth	Rio de Janeiro	239 ^b^ (49.3%)	175 ^a^ (70.3%)	**<0.001**
	Others	160 ^a^ (33%)	62 ^b^ (24.9%)	
	No response	86 ^a^ (17.7%)	12 ^b^ (4.8%)	
Level of education (n_CA_ = 386/n_CD_ = 218)	Illiterate	17 (4.4%)	2 (0.9%)	**<0.001**
	Basic	143 ^a^ (37%)	60 ^b^ (27.5%)	
	Secondary	181 (46.9%)	102 (46.8%)	
	Higher	45 ^b^ (11.7%)	54 ^a^ (24.8%)	
Income (n_CA_ = 437/n_CD_ = 236)	1 minimum salary	295 ^a^ (67.5%)	110 (46.6%)	**<0.001**
	2–3 minimum salaries	128 (29.3%)	93 ^a^ (39.4%)	
	≥4 minimum salaries	14 (3.2%)	33 ^a^ (14%)	
**General information about health**				
Suffering from allergies (yes)		173 ^b^ (35.7%)	119 ^a^ (47.8%)	**0.001**
Are you taking any medicine at the moment? (yes) (n_CA_ = 483/n_CD_ = 247)		141 ^b^ (29.2%)	106 ^a^ (42.9%)	**<0.001**
Have you ever had any rashes or itching of the skin? (yes) (n_CA_ = 484/n_CD_ = 248)		134 (27.7%)	79 (31.9%)	0.240
Have you had any rashes or itching of the skin in the last year (yes) (n_CA_ = 445/n_CD_ = 248)		91 (18.8%)	52 (21%)	0.476
Do you have a check-up every year? (yes) (n_CA_ = 445/n_CD_ = 249)		198 ^b^ (40.8%)	139 ^a^ (56.3%)	**<0.001**
Has your doctor ever diagnosed any intestinal diseases? (yes) (n_CA_ = 482/n_CD_ = 249)		60 (12.4%)	36 (14.5%)	0.446
Has your doctor ever diagnosed any skin disease)? (yes) (n_CA_ = 485/n_CD_ = 247)		41 ^b^ (8.5%)	34 ^a^ (13.8%)	**0.025**
Do you have any respiratory problems? (yes)(n_CA_ = 485/n_CD_ = 247)		89 (18.4%)	54 (21.9%)	0.257
Dengue fever (yes) (n_CA_ = 485/n_CD_ = 246)		165 (34%)	74 (30.1%)	0.283
Hepatitis A (yes)		12 (2.5%)	7 (2.8%)	0.785
Zika virus (yes) (n_CA_ = 485/n_CD_ = 249)		8 ^b^ (1.6%)	13 ^a^ (5.2%)	**0.006**
Leptospirosis (yes) (n_CA_ = 485/n_CD_ = 249)		1 (0.2%)	2 (0.8%)	0.267
Chikungunya (yes) (n_CA_ = 485/n_CD_ = 249)		10 ^b^ (2.1%)	16 ^a^ (6.4%)	**0.002**
Ascariasis/Roundworm (yes) (n_CA_ = 485/n_CD_ = 249)		54 (11.1%)	24 (9.6%)	0.534
How do you rate your physical health? (n_CA_ = 478/n_CD_ = 243)	Excellent	178 (37.2%)	90 (37%)	0.955
	Normal	267 (55.9%)	138 (56.8%)	
	Poor	27 (5.6%)	13 (5.3%)	
	Terrible	6 (1.3%)	2 (0.8%)	
How do you rate your psychological health? (n_CA_ = 463/n_CD_ = 242)	Excellent	206 ^b^ (44.5%)	139 ^a^ (57.4%)	**0.013**
	Normal	228 ^a^ (49.2%)	93 ^b^ (38.4%)	
	Poor	21 (4.5%)	7 (2.9%)	
	Terrible	8 (1.7%)	3 (1.2%)	
**Information about water and sanitation**				
The supply of water: public pipeline system (yes) (n_CA_ = 484/n_CD_ = 246)		478 (98.8%)	244 (99.2%)	0.601
Do you have a water container or tank in your house? (yes) (n_CA_ = 483/n_CD_ = 248)		450 (93.2%)	232 (93.5%)	0.845
How many litters of water do you drink every day? (n_CA_ = 474/n_CD_ = 248)	Up to 500 mL	81 (17.1%)	31 (12.5%)	0.269
	>500 mL up to <2 L	243 (51.3%)	135 (54.4%)	
	≥2 L	150 (31.6%)	82 (33.1%)	
Is this tap water? (yes) (n_CA_ = 468/n_CD_ = 242)		172 (36.8%)	96 (39.7%)	0.447
Is it bottled water (yes) (n_CA_ = 455/n_CD_ = 234)		159 (34.9%)	82 (35%)	0.980
Do you have a water filter in your house? (yes) (n_CA_ = 476/n_CD_ = 247)		398 (83.6%)	231 ^a^ (93.5%)	**<0.001**
Does the water you drink have any smell? (yes) (n_CA_ = 484/n_CD_ = 249)		59 ^a^ (12.2%)	13 (5.2%)	**0.003**
Do you use the Anil Canal water for any purposes? (yes) (n_CA_ = 485/n_CD_ = 245)		48 ^a^ (9.9%)	0 (0%)	**<0.001**
Have you ever seen/Do you sometimes see children playing in the water from Anil Canal (yes) (n_CA_ = 484/n_CD_ = 248)		364 ^a^ (75.2%)	119 ^b^ (48%)	**<0.001**
How often have you seen/do you see children playing in the Anil canal (n_CA_ = 483/n_CD_ = 248)	Every week	30 ^a^ (6.2%)	5 ^b^ (2%)	**<0.001**
	Every day	93 ^a^ (19.3%)	17 ^b^ (6.9%)	
	Occasionally	241 ^a^ (49.9%)	97 ^b^ (39.1%)	
	Never	119 ^b^ (24.6%)	129 ^a^ (52%)	
Does the water from the Anil Canal have an unpleasant smell? (yes) (n_CA_ = 483/n_CD_ = 246)		434 ^a^ (89.9%)	189 ^b^ (76.8%)	**<0.001**
How often does the water in the Anil canal have a bad smell? (n_CA_ = 444/n_CD_ = 181)	Every day	303 ^a^ (68.2%)	96 ^b^ (53%)	**<0.001**
	Once or twice a week	14 (3.2%)	13 (7.2%)	
	Occasionally	115 ^b^ (25.9%)	71 ^a^ (39.2%)	
	Never	12 (2.7%)	1 (0.6%)	
How often is the garbage collected in the locality? (n_CA_ = 470/n_CD_ = 244)	Once a week	174 (37%)	105 (43%)	0.180
	Every day	284 (60.4%)	136 (55.7%)	
	Never	12 (2.6%)	3 (1.2%)	
Is there a pavement outside your house? (yes) (n_CA_ = 470/n_CD_ = 247)		260 ^b^ (55.3%)	222 ^a^ (89.9%)	**<0.001**
Is there a gutter and sewer grate for the drainage of rainwater (yes) (n_CA_ = 471/n_CD_ = 246)		227 ^b^ (48.2%)	205 (83.3%)	**<0.001**
Do floods occur near to your residential dwelling (yes) (n_CA_ = 481/n_CD_ = 249)		426 ^a^ (88.6%)	146 ^b^ (58.6%)	**<0.001**
Do you often see serious flooding near to the Anil canal (yes) (n_CA_ = 482/n_CD_ = 248)		445 (92.3%)	165 ^b^ (66.5%)	**<0.001**
Frequency of flooding (n_CA_ = 453/n_CD_ = 180)	Always when it rains	231 (51%)	102 (56.7%)	0.397
	Sometimes	180 (39.7%)	65 (36.1%)	
	Rarely	42 (9.3%)	13 (7.2%)	
Quality of the water in the Anil canal? (n_CA_ = 472/n_CD_ = 240)	Excellent	12 (2.5%)	4 (1.7%)	**<0.001**
	Normal	75 (15.9%)	59 (24.6%)	
	Poor	53 (11.2%)	54 (22.5%)	
	Terrible	332 ^a^ (70.3%)	123 ^b^ (51.3%)	
Has the Community Agent been approached? (yes) (n_CA_ = 485/n_CD_ = 247)		361 ^a^ (74.4%)	81 ^b^ (32.8%)	**<0.001**
Frequency of contacts with the Community Agent (n_CA_ = 371/n_CD_ = 75)	Weekly	27 (7.3%)	1 (1.3%)	0.152
	Monthly	141 (38%)	31 (41.3%)	
	Once a year	203 (54.7%)	43 (57.3%)	
Do you make use of the Bárbara Mosley de Souza Family Clinic (yes) (n_CA_ = 483/n_CD_ = 249)		414 ^a^ (85.7%)	72 ^b^ (28.9%)	**<0.001**
Do you avoid making use of the Family Clinic but rely on Medical Insurance? (yes) (n_CA_ = 416/n_CD_ = 248)		52 ^b^ (12.5%)	105 ^a^ (42.3%)	**<0.001**
Does rubbish accumulate around your house? (yes) (n_CA_ = 477/n_CD_ = 248)		303 ^a^ (63.5%)	89 ^b^ (35.9%)	**<0.001**

^a,b^—these different letters indicate different meanings (^a^ indicates a group with a higher statistical ranking, ^b^ indicates a lower ranking); the values of *p* in bold indicate a significant difference/association. * The mestizo and mulatto ethnic groups are excluded from the comparison owing to their limited number. n_CA_—sample size of the Canal of Anil zone; n_CD_—sample size of the center of the district of Anil.

**Table 2:** Results and comparison of the self-perception of the quality of life (WHOQOL-bref) of the Anil Canal area and the Central District of Anil) (higher values correspond to the self-perception of a better QoL).

		Anil Canal Area	Central District of Anil	*p*
Quality of life n_CA_ = 485/n_CD_ = 249	Need to improve	22 (4.6%)	9 (3.6%)	0.391
	Normal	145 (30.1%)	65 (26.1%)	
	Good	276 (57.4%)	148 ^a^ (59.4%)	
	Very good	38 (7.9%)	27 (10.8%)	
Domain 1—Physical n_CA_ = 485/n_CD_ = 249	Median (Q1; Q3)	67.9% (57.1–78.6%)	67.9% (57.1–78.6%)	0.316
	Min-Max	12.5–100%	14.3–100%	
	Average (SD)	66.9% (16.3%)	65.8% (16.6%)	
	Need to improve	88 (18.1%)	45 (18.1%)	0.956
	Normal	222 (45.8%)	119 (47.8%)	
	Good	171 (35.3%)	83 (33.3%)	
	Very good	4 (0.8%)	2 (0.8%)	
Domain 2—Psychological n_CA_ = 485/n_CD_ = 249	Median (Q1; Q3)	75% (62.5–80%)	75% (62.5–79.2%)	0.558
	Min-Max	16.7–100%	20.8–100%	
	Average (SD)	71.5% (14.7%)	71.0% (14.3%)	
	Need to improve	39 (8%)	18 (7.2%)	0.647
	Normal	208 (42.9%)	107 (43%)	
	Good	236 (48.7%)	121 (48.6%)	
	Very good	2 (0.4%)	3 (1.2%)	
Domain 3—Relationships n_CA_ = 485/n_CD_ = 249	Median (Q1; Q3)	75% (58.3–75%)	75% (58.3–75%)	0.956
	Min-Max	0–100%	0–100%	
	Average (SD)	69% (16.3%)	68.7% (17.1%)	
	Need to improve	56 (11.6%)	28 (11.2%)	0.972
	Normal	180 (37.2%)	89 (35.7%)	
	Good	230 (47.5%)	123 (49.4%)	
	Very good	18 (3.7%)	9 (3.6%)	
Domain 4—Environment n_CA_ = 485/n_CD_ = 249	Median (Q1; Q3)	40.6% (34.4–50%)	50% (40.6–59.4%)	**<0.001**
	Min-Max	6.3–90.6%	7.1–96.9%	
	Average (SD)	42.4% (13.5%)	50.6% (14.7%)	
	Need to improve	347 ^a^ (71.5%)	107 ^b^ (43%)	**<0.001**
	Normal	126 ^b^ (26%)	132 ^a^ (53%)	
	Good	12 (2.5%)	10 (4%)	
	Very good	-	-	

^a,b^—these different letters indicate significant differences (^a^ indicates a group with a higher statistical ranking, ^b^ indicates a lower ranking); *p*-values in bold indicate a significant difference/association. n_CA_—sample size of the Canal of Anil zone; n_CD_—sample size of the center of the district of Anil.

**Table 3:** Univariate and multivariate analysis (by logistic regression) of risk/protective factors linked to the resulting “need to improve Physical QoL” in the Anil canal community and in the Central District of Anil.

		Anil Canal Area					Central District of Anil				
		QoL–Physical		*p*	OR	IC 95% OR	QoL–Physical		*p*	OR	IC 95% OR
		Need to Improve	At Least Normal				Need to Improve	At Least Normal			
**Univariate analysis of risk factors associated with the “need to improve” Physical QoL**											
Sex	F	67 (77%)	236 (59.4%)	**0.003**	1		33 (73.3%)	126 (61.8%)	0.147	1	
	M	20 (23%)	161 (40.6%)		2.285	1.334–3.914	12 (26.7%)	78 (38.2%)		1.702	0.83–3.492
Age (incremental 1 year)				**0.001**	1.025	1.01–1.041			**<0.001**	1.039	1.018–1.061
Marital status	Single	41 (50.6%)	163 (45.5%)	**<0.001**	1.000		15 (33.3%)	74 (37.4%)	0.652	1.000	
	married	24 (29.6%)	179 (50%)	**0.024**	0.533	0.309–0.921	23 (51.1%)	89 (44.9%)	0.508	1.275	0.621–2.619
	divorced	2 (2.5%)	10 (2.8%)	0.773	0.795	0.168–3.77	2 (4.4%)	18 (9.1%)	0.451	0.548	0.115–2.616
	Widowed	14 (17.3%)	6 (1.7%)	**<0.001**	9.276	3.359–25.618	5 (11.1%)	17 (8.6%)	0.523	1.451	0.464–4.542
Level of education	Illiterate	6 (10.7%)	11 (3.3%)	**0.002**	1.000		1 (2.6%)	1 (0.6%)	0.458	1.000	
	basic	30 (53.6%)	113 (34.2%)	**0.034**	4.364	1.118–17.028	10 (25.6%)	50 (27.9%)	0.196	6.714	0.376–120.006
	secondary school	15 (26.8%)	166 (50.3%)	0.145	2.124	0.771–5.849	21 (53.8%)	81 (45.3%)	0.580	1.343	0.472–3.817
	Higher	5 (8.9%)	40 (12.1%)	0.552	0.723	0.248–2.106	7 (17.9%)	47 (26.3%)	0.242	1.741	0.688–4.402
Income	≥4 m.s.	1 (1.3%)	13 (3.6%)	0.070	1.000		4 (9.5%)	29 (14.9%)	0.448	1	
	1 m.s.	62 (78.5%)	233 (65.1%)	0.236	3.459	0.444–26.956	23 (54.8%)	87 (44.8%)	0.264	1.917	0.612–6.005
	2–3 m.s.	16 (20.3%)	112 (31.3%)	0.563	1.857	0.227–15.171	15 (35.7%)	78 (40.2%)	0.582	1.394	0.427–4.548
Suffering from allergies	No	46 (52.3%)	266 (67%)	**0.010**	1		24 (53.3%)	106 (52%)	0.868	1	
	Yes	42 (47.7%)	131 (33%)		1.854	1.161–2.96	21 (46.7%)	98 (48%)		0.946	0.496–1.807
Currently taking medicine	No	45 (51.1%)	297 (75.2%)	**<0.001**	1		13 (29.5%)	128 (63.1%)	**<0.001**	1	
	Yes	43 (48.9%)	98 (24.8%)		2.896	1.799–4.663	31 (70.5%)	75 (36.9%)		4.070	2.006–8.258
Has your doctor diagnosed any intestinal infections?	No	72 (81.8%)	350 (88.8%)	0.074	1		36 (80%)	177 (86.8%)	0.246	1	
	Yes	16 (18.2%)	44 (11.2%)		1.768	0.945–3.305	9 (20%)	27 (13.2%)		1.639	0.711–3.778
Has your doctor diagnosed any skin diseases?	No	81 (92%)	363 (91.4%)	0.852	1		35 (77.8%)	178 (88.1%)	0.073	1	
	Yes	7 (8%)	34 (8.6%)		0.923	0.395–2.155	10 (22.2%)	24 (11.9%)		2.119	0.932–4.821
Do you have any respiratory problems?	No	59 (67%)	337 (84.9%)	**<0.001**	1		32 (72.7%)	161 (79.3%)	0.340	1	
	Yes	29 (33%)	60 (15.1%)		2.761	1.637–4.655	12 (27.3%)	42 (20.7%)		1.437	0.682–3.029
Dengue fever	No	48 (54.5%)	272 (68.5%)	**0.013**	1		29 (64.4%)	143 (71.1%)	0.377	1	
	Yes	40 (45.5%)	125 (31.5%)		1.813	1.133–2.901	16 (35.6%)	58 (28.9%)		1.360	0.688–2.691
Hepatitis A	No	88 (100%)	385 (97%)	n.a.			42 (93.3%)	200 (98%)	0.104	1	
	Yes	0 (0%)	12 (3%)				3 (6.7%)	4 (2%)		3.571	0.771–16.551
Do you have a water container or tank in your house?	Sim	7 (8%)	26 (6.6%)	0.645	1		5 (11.1%)	11 (5.4%)	0.169	1	
	No	81 (92%)	369 (93.4%)		0.815	0.342–1.943	40 (88.9%)	192 (94.6%)		0.458	0.151–1.392
Do you have filtered water in your house?	Yes	63 (73.3%)	335 (85.9%)	**0.005**	1		39 (88.6%)	192 (94.6%)	0.156	1	
	No	23 (26.7%)	55 (14.1%)		2.224	1.275–3.878	5 (11.4%)	11 (5.4%)		2.238	0.736–6.802
Does the water you drink have any smell?	Yes	71 (80.7%)	354 (89.4%)	**0.026**	1		41 (91.1%)	195 (95.6%)	0.231	1	
	No	17 (19.3%)	42 (10.6%)		2.018	1.087–3.745	4 (8.9%)	9 (4.4%)		2.114	0.621–7.196
Do you use the Anil Canal water any activities?	No	73 (83%)	364 (91.7%)	**0.015**	1						
	Yes	15 (17%)	33 (8.3%)		2.267	1.171–4.385					
Does the Anil Canal water have an unpleasant smell?	No	84 (97.7%)	350 (88.2%)	**0.018**	1		34 (75.6%)	155 (77.1%)	0.823	1	
	Yes	2 (2.3%)	47 (11.8%)		5.640	1.343–23.686	11 (24.4%)	46 (22.9%)		0.917	0.431–1.952
**Using adjustment in multivariate analysis** **^†^ (by logistic regression) of risk factors associated with “the need to improve” the physical QoL**											
Age									**0.023**	1.034	1.005–1.064
Marital Status	Single			**<0.001**							
	Married			**0.043**	0.526	0.282–0.980					
	Divorced			0.794	0.799	0.149–4.300					
	Widowed			**<0.001**	11.414	3.770–34.554					
Level of education	Illiterate								0.075		
	Basic								0.373	4.082	0.185–89.917
	secondary								0.178	2.511	0.659–9.569
	Higher								**0.011**	5.336	1.458–19.525
Are you currently taking any medicine? yes				**<0.001**	3.198	1.769–5.781			**0.013**	3.419	1.300–8.993
Do you have any respiratory problems? yes				**<0.001**	3.587	1.886–6.822					
Has your doctor ever diagnosed any skin diseases? yes									**0.002**	6.490	1.989–21.181
Do you have a water container/tank in your house? yes									**0.042**	0.192	0.039–0.944
Do you have filtered water in your house? yes				**<0.001**	3.299	1.704–6.386			0.059	4.011	0.951–16.914
Does the Anil Canal have an unpleasant smell? yes				**0.041**	4.898	1.064–22.544					
Constant				**<0.001**	0.020				**0.001**	0.023	
Quality of the Model:		% of the correct responses predicted by this model: 84.3%; AUC = 0.805 (IC 95%: 0.75–0.86); R^2^_Cox & Snell_ = 0.174; R^2^_Nagelkerke_ = 0.284; Hosmer and Lemeshaw test: *p* = 0904					% of the correct responses predicted by this model: 84.8%; AUC = 0.0801 (IC 95%: 0.73–0.88); R^2^_Cox & Snell_ = 0.183; R^2^_Nagelkerke_ = 0.289; Hosmer and Lemeshaw test: *p* = 0.571				
Variables that were introduced into the model–initial stage.		Sex, Age, Marital Status, Level of Education, Allergies? Are you currently taking any medicine? Has your doctor ever diagnosed any intestinal diseases)? Has your doctor ever diagnosed any skin disease? Do you haver any respiratory problems? Dengue fever? Hepatitis A? Do you have a water container or tank in your house? Is this water tap water? Do you have a water filter in your house? Does the water you drink have an unpleasant smell? Do you use water from the Anil Canal for any activities? Does the water from the Anil Canal have an unpleasant smell?					Sex, Ethnicity, Marital Status, Level of Education, Income? Have you experienced any rashes or itching on your skin in the last year? Has your doctor ever diagnosed any intestinal diseases)? Ascariasis/roundworm. Do you have a water container or tank in your house? Is the water tap water? Is it bottled water? Have you got a water filter in your house? Does the water you drink have a bad smell? Does the Anil Canal water have an unpleasant smell? Does the street in front of your house have a pavement? Is there a gutter and sewer grate for draining the rainwater? Do floods occur near to your residential dwelling? Do you often see serious floods in the area close to the Anil Canal? Quality of water in the Anil Canal? Does garbage accumulate around your house?				

OR: Odds ratio; IC 95% for OR: Confidence Interval of 95% for the Odds ratio; n.a.—not-applicable owing to a lack of observations/counts. The values of *p* in bold indicate a significant link with the “need to improve the Physical Domain”. † Analysis adjusted for the marital status and schooling categories.

**Table 4:** Univariate and multivariate analysis (by logistic regression) of the risk/protective factors that are entailed in the resulting “need to improve” the QoL environment in the Anil Canal community and in the Central District of Anil.

		Anil Canal Area					Central District of Anil				
		QoL–Environment		*p*	OR	IC 95% OR	QoL–Environment		*p*	OR	IC 95% OR
		Need to Improve	At Least Normal				Need to Improve	At Least Normal			
**Univariate analysis of risk factors associated with the “need to improve” environmental QoL**											
Sex	F	226 (65.3%)	77 (55.8%)	0.051	1.492	0.998–2.231	73 (68.2%)	86 (60.6%)	0.214	1.398	0.824–2.371
	M	120 (34.7%)	61 (44.2%)		1		34 (31.8%)	56 (39.4%)		1	
Age (incremental 1 year)				0.484	1.005	0.992–1.018			0.978	1.000	0.985–1.015
Ethnicity *	white	53 (21.7%)	30 (31.6%)	0.169	1		25 (30.5%)	63 (53.8%)	**0.032**	1	
	black	33 (13.5%)	11 (11.6%)	0.204	1.698	0.751–3.841	10 (12.2%)	14 (12%)	0.218	1.800	0.707–4.582
	brown	158 (64.8%)	54 (56.8%)	0.069	1.656	0.961–2.854	45 (54.9%)	40 (34.2%)	**0.001**	2.835	1.511–5.319
Marital status	single	157 (50%)	47 (37.6%)	0.055	1		45 (43.7%)	44 (31.4%)	0.084	1	
	married	136 (43.3%)	67 (53.6%)	**0.026**	0.608	0.392–0.942	44 (42.7%)	68 (48.6%)	0.111	0.633	0.36–1.11
	divorced	6 (1.9%)	6 (4.8%)	**0.045**	0.299	0.092–0.972	4 (3.9%)	16 (11.4%)	**0.018**	0.244	0.076–0.789
	Widowed	15 (4.8%)	5 (4%)	0.843	0.898	0.31–2.601	10 (9.7%)	12 (8.6%)	0.668	0.815	0.319–2.079
Level of education	Illiterate	14 (5.1%)	3 (2.7%)	0.061	3.733	0.941–14.819	2 (2.3%)	0 (0%)	n.a.		
	Basic	111 (40.7%)	32 (28.3%)	**0.005**	2.775	1.368–5.63	32 (36.4%)	28 (21.5%)	**0.002**	3.604	1.613–8.054
	Secondary	123 (45.1%)	58 (51.3%)	0.120	1.697	0.872–3.301	41 (46.6%)	61 (46.9%)	**0.046**	2.120	1.013–4.438
	Higher	25 (9.2%)	20 (17.7%)	**0.021**	1		13 (14.8%)	41 (31.5%)	**0.021**	1	
Income	1 m.s.	225 (72.6%)	70 (55.1%)	**<0.001**	11.786	3.198–43.439	58 (58%)	52 (38.2%)	**<0.001**	6.246	2.247–17.366
	2–3 m.s.	82 (26.5%)	46 (36.2%)	**0.006**	6.536	1.734–24.632	37 (37%)	56 (41.2%)	**0.014**	3.700	1.31–10.45
	≥4 m.s.	3 (1%)	11 (8.7%)	**<0.001**	1		5 (5%)	28 (20.6%)	**0.001**	1	
Have you ever had a rash or itching on the skin?	No	251 (72.3%)	99 (72.3%)	0.987	0.996	0.641–1.55	80 (74.8%)	89 (63.1%)	0.052	0.578	0.332–1.006
	Yes	96 (27.7%)	38 (27.7%)		1		27 (25.2%)	52 (36.9%)		1	
Have you had a rash or itching on the skin in the last year?	No	290 (83.6%)	104 (75.4%)	**0.038**	1.663	1.029–2.688	88 (82.2%)	108 (76.6%)	0.281	1.415	0.753–2.659
	Yes	57 (16.4%)	34 (24.6%)		1		19 (17.8%)	33 (23.4%)		1	
Do you have a check-up every year?	No	220 (63.4%)	67 (48.6%)	**0.003**	1.836	1.232–2.736	55 (51.4%)	53 (37.9%)	**0.034**	1.736	1.042–2.892
	Yes	127 (36.6%)	71 (51.4%)		1		52 (48.6%)	87 (62.1%)		1	
Has your doctor ever diagnosed any intestinal diseases?	No	307 (89%)	115 (83.9%)	0.132	1.546	0.877–2.725	91 (85%)	122 (85.9%)	0.847	0.932	0.458–1.899
	Yes	38 (11%)	22 (16.1%)		1		16 (15%)	20 (14.1%)		1	
Hepatitis A?	No	338 (97.4%)	135 (97.8%)	0.789	0.835	0.223–3.13	102 (95.3%)	140 (98.6%)	0.145	0.291	0.055–1.532
	Yes	9 (2.6%)	3 (2.2%)		1		5 (4.7%)	2 (1.4%)		1	
Zika virus?	No	344 (99.1%)	133 (96.4%)	**0.048**	4.311	1.016–18.291	105 (98.1%)	131 (92.3%)	0.057	4.408	0.956–20.325
	Yes	3 (0.9%)	5 (3.6%)		1		2 (1.9%)	11 (7.7%)		1	
Chikungunya?	No	339 (97.7%)	136 (98.6%)	0.553	0.623	0.131–2.972	103 (96.3%)	130 (91.5%)	0.144	2.377	0.745–7.588
	Yes	8 (2.3%)	2 (1.4%)		1		4 (3.7%)	12 (8.5%)		1	
Ascariasis/Roundworm	No	317 (91.4%)	114 (82.6%)	**0.007**	2.225	1.248–3.965	98 (91.6%)	127 (89.4%)	0.570	1.286	0.54–3.062
	Yes	30 (8.6%)	24 (17.4%)		1		9 (8.4%)	15 (10.6%)		1	
Do you have a water container or tank in your house?	Yes	326 (94.2%)	124 (90.5%)	0.149	1.709	0.825–3.536	103 (96.3%)	129 (91.5%)	0.140	2.398	0.750–7.633
	No	20 (5.8%)	13 (9.5%)		1		4 (3.7%)	12 (8.5%)		1	
Do you drink tap water?	No	205 (61%)	91 (68.9%)	0.110	0.705	0.459–1.083	61 (58.7%)	85 (61.6%)	0.644	0.885	0.526–1.487
	Yes	131 (39%)	41 (31.1%)		1		43 (41.3%)	53 (38.4%)		1	
Do you drink bottled water?	No	220 (67.5%)	76 (58.9%)	0.085	1.447	0.951–2.204	62 (63.3%)	90 (66.2%)	0.645	0.880	0.511–1.515
	Yes	106 (32.5%)	53 (41.1%)		1		36 (36.7%)	46 (33.8%)		1	
Do you drink filtered water in your house?	No	65 (19%)	13 (9.7%)	**0.016**	2.184	1.16–4.112	10 (9.3%)	6 (4.3%)	0.118	2.302	0.809–6.549
	Yes	277 (81%)	121 (90.3%)		1		97 (90.7%)	134 (95.7%)		1	
Does the water you drink have a bad smell?	Yes	47 (13.6%)	12 (8.7%)	0.141	1.651	0.847–3.217	10 (9.3%)	3 (2.1%)	**0.020**	4.777	1.281–17.81
	No	299 (86.4%)	126 (91.3%)		1		97 (90.7%)	139 (97.9%)		1	
Does the Anil Canal water have an unpleasant smell?	Yes	316 (91.6%)	118 (85.5%)	**0.048**	1.847	1.006–3.391	85 (81%)	104 (73.8%)	0.188	1.512	0.818–2.796
	No	29 (8.4%)	20 (14.5%)		1		20 (19%)	37 (26.2%)		1	
How often does the Anil Canal water have an unpleasant smell?	Every day	231 (71.5%)	72 (59.5%)	0.061	1		54 (64.3%)	42 (43.3%)	**0.038**	1	
	1–2/week	7 (2.2%)	7 (5.8%)	**0.034**	0.312	0.106–0.918	4 (4.8%)	9 (9.3%)	0.094	0.346	0.1–1.2
	Occasionally	77 (23.8%)	38 (31.4%)	**0.055**	0.632	0.395–1.011	25 (29.8%)	46 (47.4%)	**0.008**	0.423	0.225–0.795
	Never	8 (2.5%)	4 (3.3%)	0.451	0.623	0.182–2.131	1 (1.2%)	0 (0%)	n.a.		
Is there a pavement in the street outside your house?	No	174 (51.3%)	36 (27.5%)	**<0.001**	2.783	1.794–4.316	14 (13.3%)	11 (7.7%)	0.155	1.832	0.796–4.218
	Yes	165 (48.7%)	95 (72.5%)		1		91 (86.7%)	131 (92.3%)		1	
Is there a gutter and sewer grate for draining rainwater?	No	188 (56.3%)	56 (40.9%)	**0.003**	1.863	1.244–2.788	24 (23.1%)	17 (12%)	**0.023**	2.206	1.116–4.362
	Yes	146 (43.7%)	81 (59.1%)		1		80 (76.9%)	125 (88%)		1	
Do floods occur near to your residential dwelling?	Yes	317 (92.2%)	109 (79.6%)	**<0.001**	3.016	1.703–5.342	74 (69.2%)	72 (50.7%)	**0.004**	2.180	1.289–3.689
	No	27 (7.8%)	28 (20.4%)		1		33 (30.8%)	70 (49.3%)		1	
Do you often see serious floods close to the Anil Canal?	No	18 (5.2%)	19 (13.9%)	**0.002**	1		30 (28%)	53 (37.6%)	0.115	1	
	Yes	327 (94.8%)	118 (86.1%)		2.924	1.484–5.747	77 (72%)	88 (62.4%)		1.546	0.898–2.660
Frequency of flooding in the Anil Canal	Whenever it rains	172 (51.8%)	59 (48.8%)	**0.005**	2.650	1.351–5.199	55 (62.5%)	47 (51.1%)	0.598	1.365	0.429–4.346
	sometimes	138 (41.6%)	42 (34.7%)	**0.002**	2.987	1.488–5.998	27 (30.7%)	38 (41.3%)	0.759	0.829	0.25–2.743
	Rarely	22 (6.6%)	20 (16.5%)	**0.007**	1		6 (6.8%)	7 (7.6%)	0.292	1	
Quality of the water in the Anil Canal	Excellent	10 (2.9%)	2 (1.5%)	0.489	1.721	0.37–8.011	4 (3.8%)	0 (0%)	n.a.		
	Normal	41 (12%)	34 (26%)	**0.001**	0.415	0.247–0.696	23 (22.1%)	36 (26.5%)	0.255	0.693	0.369–1.303
	Poor	43 (12.6%)	10 (7.6%)	0.293	1.480	0.712–3.073	18 (17.3%)	36 (26.5%)	0.072	0.542	0.278–1.057
	Terrible	247 (72.4%)	85 (64.9%)	**0.002**	1		59 (56.7%)	64 (47.1%)	0.304	1	
Frequency of contact with the Community Agent	Weekly	16 (6.1%)	11 (10.2%)	0.091	0.488	0.213–1.12	0 (0%)	1 (2.6%)	n.a.		
	Monthly	95 (36.1%)	46 (42.6%)	0.129	0.693	0.431–1.113	13 (36.1%)	18 (46.2%)	0.328	0.628	0.247–1.594
	Once a year	152 (57.8%)	51 (47.2%)	0.127	1		23 (63.9%)	20 (51.3%)	0.619	1	
Does garbage accumulate around your house?	No	104 (30.5%)	70 (51.5%)	**<0.001**	1		51 (48.1%)	108 (76.1%)	**<0.001**	1	
	Yes	237 (69.5%)	66 (48.5%)		2.417	1.608–3.634	55 (51.9%)	34 (23.9%)		3.426	1.992–5.890
**Adjusted Multivariate analysis † (by logistic regression) of the risk factors entailed in the “need to improve” the Environmental QoL**											
Income. Reference-point: ≥4 m.s.				**0.006**	1				**0.004**	1	
Income = 1 m.s.				**0.003**	10.225	2.246–46.544			**0.002**	5.629	1.920–16.508
Income? 2–3 m.s.				**0.016**	6.659	1.434–30.931			**0.033**	3.287	1.102–9.801
Ascariasis/Roundworm: have not had this				**0.019**	2.465	1.163–5.227					
Do you have a water container/tank in your house? Yes				**0.020**	3.101	1.194–8.054					
Do you drink bottled water? No				**0.011**	2.081	1.184–3.658					
Does the street outside your house have a pavement? No				**0.013**	2.082	1.166–3.719					
Is there a gutter and sewer grate for draining rainwater? No									0.138	1.803	0.827–3.930
Does garbage accumulate around your house? Yes				**0.001**	2.601	1.509–4.483			**<0.001**	2.960	1.652–5.304
Constant				**<0.001**	0.013				**<0.001**	0.117	
Quality of the model:		% of correct responses predicted by this model: 76.6%; AUC = 0.75 (IC95%: 0.70–0.81); R^2^_Cox & Snell_ = 0.175; R^2^_Nagelkerke_ = 0.248; Hosmer and Lemeshaw test: *p* = 0.772					% of correct responses predicted by this model: 67%; AUC = 0.72 (IC95%: 0.66–0.79); R^2^_Cox & Snell_ = 0.138; R^2^_Nagelkerke_ = 0.186; Hosmer and Lemeshaw test: *p* = 0.771				
Variables that are introduced into the model–initial stage		Sex, Ethnicity, Marital Status, Level of Education, Income, Have you had a rash or itching on the skin in the last year? Has your doctor diagnosed any intestinal diseases, Ascariasis/Roundworm. Do you have a water container or tank in your house? Is this tap water? Is this bottled water? Do have a water filter in your house? Does the water you drink have an unpleasant smell? Does the Anil Canal water have an unpleasant smell? Is there a pavement in the street outside your house? Is there a gutter and sewer grate for draining the rainwater? Do floods occur near your residential dwelling? Do you often see serious floods in the area close to the Anil Canal? What is the quality of water like in the Anil Canal? Does rubbish accumulate around your house?					Ethnicity, Marital Status, Level of Education? Have you ever suffered from rashes or itching of the skin? Do you have a medical checkup every year? Hepatitis A? Zika? Do you have a water container or tank in your house? Is the water bottled? Do you have a water filter in the house? Does the water you drink have a smell? Does the Anil Canal water have a bad smell? Does the street outside your house have a pavement? Is there a gutter and sewer grate for draining the rainwater? Do floods occur near to your residential dwelling? Do you often see serious floods close to the area of the Anil Canal? What is the quality of Anil Canal water like? Does rubbish accumulate around your house?				

OR: Odds Ratio; IC of 95% for OR: Confidence Interval of 95% for the Odds Ratio; n.a.—not applicable because of a lack if observations/counts. * The mestizo and mulatto ethnic groups are not included in the analysis. The values of *p* in bold indicate a significant association with the “need to improve” environment domain. † Analysis adjusted for marital status and schooling.

## Data Availability

The data presented in this study are available on request from the corresponding author, although raw data will not be shared due to privacy restrictions of the participants.
